# Nomogram for predicting the preoperative lymph node metastasis in resectable pancreatic cancer

**DOI:** 10.1007/s00432-023-05048-8

**Published:** 2023-07-14

**Authors:** Hao Cheng, Jin-Hong Xu, Xiao-Hong Kang, Xiao-Mei Liu, Hai-Feng Wang, Zhi-Xia Wang, Hao-Qi Pan, Qing-Qin Zhang, Xue-Lian Xu

**Affiliations:** 1 Department of Oncology, The First Affiliated Hospital of Xinxiang Medical University, 88 Jiankang Road, Xinxiang, 453100 Henan China; 2 Department of Otolaryngology, AnYang District Hospital, Anyang, 455000 Henan China; 3 Department of Respiratory Medicine, The First Affiliated Hospital of Xinxiang Medical University, Xinxiang, 453100 Henan China

**Keywords:** Nomogram, Resectable pancreatic cancer, Lymph node metastasis, Preoperative, SEER

## Abstract

**Background:**

Lymph node metastasis (LNM) is a critical prognostic factor in resectable pancreatic cancer (PC) patients, determining treatment strategies. This study aimed to develop a clinical model to adequately and accurately predict the risk of LNM in PC patients.

**Methods:**

13,200 resectable PC patients were enrolled from the SEER (Surveillance, Epidemiology, and End Results) database, and randomly divided into a training group and an internal validation group at a ratio of 7:3. An independent group (*n* = 62) obtained from The First Affiliated Hospital of Xinxiang Medical University was enrolled as the external validation group. The univariate and multivariate logistic regression analyses were used to screen independent risk factors for LNM. The minimum Akaike’s information criterion (AIC) was performed to select the optimal model parameters and construct a nomogram for assessing the risk of LNM. The performance of the nomogram was assessed by the receiver operating characteristics (ROC) curve, calibration plot, and decision curve analysis (DCA). In addition, an online web calculator was designed to assess the risk of LNM.

**Result:**

A total of six risk predictors (including age at diagnosis, race, primary site, grade, histology, and T-stage) were identified and included in the nomogram. The areas under the curves (AUCs) [95% confidential interval (CI)] were 0.711 (95%CI: 0.700–0.722), 0.700 (95%CI: 0.683–0.717), and 0.845 (95%CI: 0.749–0.942) in the training, internal validation and external validation groups, respectively. The calibration curves showed satisfied consistency between nomogram-predicted LNM and actual observed LNM. The concordance indexes (C-indexes) in the training, internal, and external validation sets were 0.689, 0.686, and 0.752, respectively. The DCA curves of the nomogram demonstrated good clinical utility.

**Conclusion:**

We constructed a nomogram model for predicting LNM in pancreatic cancer patients, which may help oncologists and surgeons to choose more individualized clinical treatment strategies and make better clinical decisions.

## Introduction

PC is the most aggressive and lethal malignancy in gastrointestinal cancers. The overall 5-year survival rate is less than 10%, with few significant improvements for years (Ansari et al. 2016; Siegel et al. 2022). The primary treatment for PC including surgery, neoadjuvant therapy, and postoperative therapy, surgical resection is considered to be the only potentially curative treatment among those treatments (Stott et al. 2022). However, most PC patients underwent surgical resection with inadequate number and extent of lymph node dissection (Groot et al. 2017; Kovac et al. 2019). Mostly, it is difficult to get R0 excision and patients diagnosed with PC usually experience early local recurrence and metastasis after surgery (Suto et al. 2022; Torphy et al. 2020). Besides, it is insufficient to evaluate the preoperative LNM on the imaging appearance solely. Therefore, the evaluation of preoperative LNM is an important prognostic determinant factor for resectable PC, which determined the surgical resection type and the implementation of preoperative neoadjuvant therapy and aggressive postoperative adjuvant therapy (Shi et al. 2019; Suto et al. 2022).

The nomogram model has been widely used in the prediction of lymph node metastasis. However, there is a lack of nomograms for predicting LNM in resectable PC patients preoperatively. In this study, the clinical characteristics of cases diagnosed with PC were analyzed, and a nomogram for predicting LNM was developed, which contributes to providing personalized guidance for resectable PC patients.

## Materials and methods

### Data collection

**Fig. 1:**
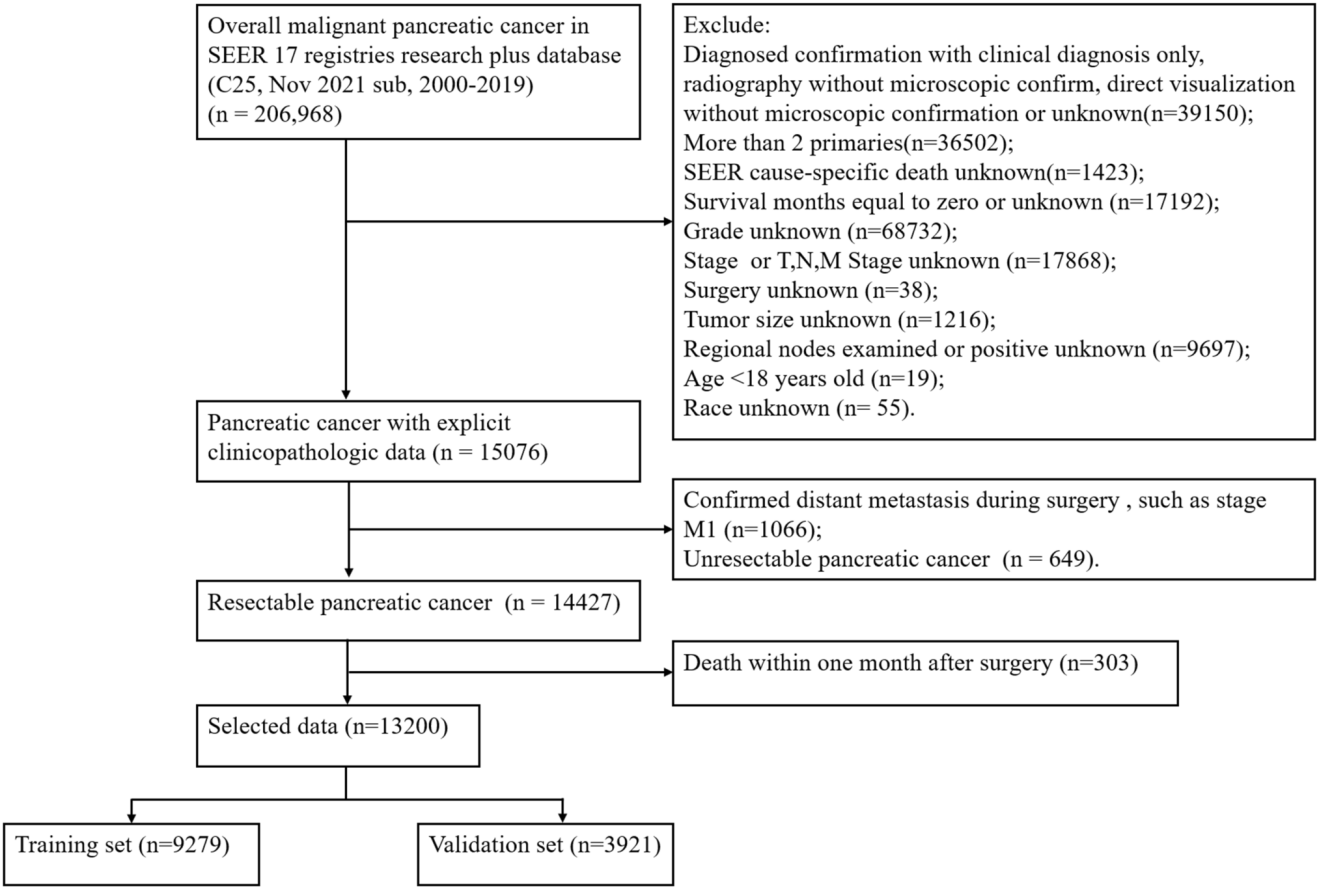
Patients enrollment and exclusion process in the SEER database

In our study, patients diagnosed with pancreatic cancer from 2000 to 2019 were collected from the SEER database. The exclusion criteria were as follows: diagnosed confirmation with clinical diagnosis only, radiography without microscopic confirm, direct visualization without microscopic confirmation or unknown; more than 2 primaries; SEER cause-specific death unknown; survival months equal to zero or unknown; grade unknown; stage or *T*, *N*, *M* Stage unknown; surgery unknown; tumor size unknown; regional nodes examined or positive unknown; age < 18 years old; race unknown; confirmed distant metastasis during surgery (stage M1); unresectable pancreatic cancer; death within one month after surgery. The following clinicopathological variables of gender, age at diagnosis, race, grade, primary site, histology, T-stage, and lymph node status were collected. The screening flowchart is shown in Fig. 1.

A total of 62 patients diagnosed with PC from December 2018 to February 2022 in The First Affiliated Hospital of Xinxiang Medical University were used to further validate the constructed nomogram externally. The inclusion and exclusion criteria were the same as the training set. The time of the last follow-up was March 2023. This study was approved by the institutional review board of our hospital.

### Statistical analysis

The median (IQR), frequency (proportions), Mann–Whitney U tests, independent t-tests, Pearson’s chi-square test, Fisher’s exact test, and univariate and multivariate binary logistic regression analysis were calculated by SPSS (SPSS Inc., Chicago, USA). Nomograms, ROC curves, calibration plots, the nutrition risk index (NRI), integrated discrimination improvement (IDI), DCA curves, and Kaplan–Meier plots, were conducted by R software (version 4.2.2). *P* < 0.05 was considered statistically significant.

### Construction, validation, and clinical usefulness of the nomogram

Univariate and multivariate logistic regression analyses were utilized to find the independent factors in predicting LNM and the minimum Akaike’s information criterion (AIC) was performed to choose the optimal model parameters and construct a nomogram for evaluating the risk of LNM. The predictors include age at diagnosis, race, primary site, grade, histology, and *T*-stage. Nomogram was constructed based on these variables (a dynamic nomogram was also provided in our study). The accuracy and discrimination of the nomogram were assessed by the ROC curve and the C-index. The calibration curves were utilized to evaluate the consistency between the actual outcomes and the predicted probabilities. The NRI and IDI were calculated to compare the performance between the nomogram and the clinical predictors. Additionally, the clinical utility in decision-making was assessed by DCA.

## Results

### Patient characteristics

A total of 13,200 resectable pancreatic cancer patients were enrolled in our research between 2000 and 2019 according to the screening flowchart from the SEER database and randomly divided into a training group (*n* = 9279) and internal validation group (*n* = 3921) at a ratio of 7:3 (Fig. 1). Meanwhile, 62 patients who underwent surgical resection with PC were obtained from the First Affiliated Hospital of Xinxiang Medical University and applied as the external validation group. The detailed clinicopathological features of all patients are presented in Table 1. There was no significant difference in the three groups except the race (*P* < 0.001) (Table 1).

**Table 1:** Clinicopathological characteristics in resectable pancreatic cancer

Characteristics	Total (*N*, %)	Training (*N*, %)	Internal validation (*N*, %)	External validation (N, %)	P value
Age [median (IQR)]	65 (57,73)	65 (57,73)	65 (57,73)	61 (55–64)	0.531
Race					< 0.001
White	10,713 (81.2)	7541 (81.3)	3172 (80.9)	0	
Black	1267 (9.6%)	889 (9.6)	378 (9.6)	0	
Asian	1153 (8.7)	804 (8.7)	349 (8.9%)	62 (100%)	
AI/AN	67 (0.5)	45 (0.5%)	22 (0.6%)	0	
Sex					0.957
Female	6429 (48.7%)	4525 (48.8%)	1904 (48.6%)	31 (50.0%)	
Male	6771 (51.3%)	4754 (51.2%)	2017 (51.4%)	31 (50.0%)	
Primary site					0.214
Head of pancreas	9103 (69.0%)	6413 (69.1%)	2690 (68.6%)	34 (54.8%)	
Body of pancreas	1038 (7.9%)	728 (7.8%)	310 (7.9%)	6 (9.7%)	
Tail of pancreas	1755 (13.3%)	1247 (13.4%)	508 (13.0%)	17 (27.4%)	
Pancreatic duct	118 (0.9%)	81 (0.9%)	37 (0.9%)	1 (1.6%)	
Others^a^	1186 (9.0%)	810 (8.7%)	376 (9.6%)	4 (6.5%)	
Grade					0.358
G1	2606 (19.7%)	1832 (19.7%)	774 (19.7%)	6 (9.7%)	
G2	6270 (47.5%)	4404 (47.5%)	1866 (47.6%)	35 (56.5%)	
G3	4140 (31.4%)	2913 (31.4%)	1227 (31.3%)	14 (22.6%)	
G4	184 (1.4%)	130 (1.4%)	54 (1.4%)	7 (11.3%)	
Histology					0.095
Adenocarcinoma	5767 (43.7%)	4092 (44.1%)	1675 (42.7%)	22 (35.5%)	
Infiltrating duct carcinoma	4680 (35.5%)	3269 (35.2%)	1411 (36.0%)	17 (27.4%)	
Neuroendocrine carcinoma	739 (5.6%)	493 (5.3%)	246 (6.3%)	9 (14.5%)	
Others^b^	2014 (15.3%)	1425 (15.4%)	589 (15.0%)	14 (22.6%)	
T-Stage					0.457
T1	1313 (9.9%)	906 (9.8%)	407 (10.4%)	7 (11.3%)	
T2	2864 (21.7%)	2507 (22.2%)	807 (20.6%)	33 (53.2%)	
T3	8503 (64.4%)	5958 (64.2%)	2545 (64.9%)	16 (25.8%)	
T4	520 (3.9%)	358 (3.9%)	162 (4.1%)	6 (9.7%)	

Note: *IQR* Inter-quartile range; Asian, Asian or Pacific Islander; *AI/AN* American Indian/Alaska Native

Others^a^, Other specified parts of pancreas, Islets of Langerhans, Overlapping lesion of pancreas and Pancreas, NOS

Others^b^, Squamous cell carcinoma, Pancreatic endocrine tumor, Atypical carcinoid tumor, etc

### Univariate and multivariate logistic regression results

The clinicopathological factors associated with LNM were revealed by the univariate and multivariate logistic regression analysis. Univariate logistic regression analysis showed that age at diagnosis, race, primary site, grade, histology, and T-stage were significant factors for LNM in PC patients (Table 2). Consequently, we figured out independent factors by multivariate logistic regression analysis, including race [Asian: odds ratio (OR) = 0.807 (95%CI = 0.686–0.949), *P* = 0.009], primary site [Body of pancreas: OR = 0.479 (95%CI = 0.404–0.568), *P* < 0.001], grade [G3: OR = 1.904 (95%CI = 1.642–2.208), *P* < 0.001], histology [Neuroendocrine carcinoma: OR = 5.465 (95%CI = 4.586–6.513), *P* < 0.001], and T-stage [T4: OR = 4.892 (95%CI = 3.694–6.408), *P* < 0.001] (Table 2).

**Table 2:** Risk variables for lymph node metastasis determined by univariate and multivariate logistic regression analyses

Variables	Univariate analysis		Multivariate analysis	
	OR (95% CI)	*P*	OR (95% CI)	*P*
Age	1.010 (1.007–1.014)	< 0.001	0.996 (0.992–1.000)	0.077
Race				
White	Reference		Reference	
Black	0.968 (0.838–1.117)	0.653	1.009 (0.937–1.288)	0.246
Asian	0.755 (0.652–0.874)	< 0.001	0.807 (0.686–0.949)	0.009
AI/AN	0.968 (0.529–1.772)	0.917	1.204 (0.621–2.332)	0.583
Sex				
Female	Reference			
Male	1.054 (0.969–1.146)	0.219		
Primary site				
Head of pancreas	Reference		Reference	
Body of pancreas	0.334 (0.286–0.391)	< 0.001	0.479 (0.404–0.568)	< 0.001
Tail of pancreas	0.296 (0.261–0.335)	< 0.001	0.477 (0.415–0.549)	< 0.001
Pancreatic duct	0.703 (0.448–1.104)	0.126	0.722 (0.447–1.165)	0.182
Others^a^	0.534 (0.461–0.620)	< 0.001	0.744 (0.632–0.876)	< 0.001
Grade				
G1	Reference		Reference	
G2	3.074 (2.746–3.441)	< 0.001	1.554 (1.356–1.781)	< 0.001
G3	4.210 (3.718–4.768)	< 0.001	1.904 (1.642–2.208)	< 0.001
G4	2.052 (1.433–2.938)	< 0.001	1.403 (0.957–2.057)	0.083
Histology				
Adenocarcinoma	Reference		Reference	
Infiltrating duct carcinoma	1.211 (1.095–1.338)	< 0.001	2.857 (2.370–3.455)	< 0.001
Neuroendocrine carcinoma	0.215 (0.176–0.263)	< 0.001	5.465 (4.586–6.513)	< 0.001
Others^b^	0.286 (0.253–0.325)	< 0.001	4.892 (3.694–6.480)	< 0.001
*T*_Stage				
T1	Reference		Reference	
T2	3.535 (2.960–4.222)	< 0.001	2.857 (2.370–3.445)	< 0.001
T3	8.627 (7.315–10.176)	< 0.001	5.465 (4.586–6.513)	< 0.001
T4	6.956 (5.311–9.110)	< 0.001	4.892 (3.694–6.480)	< 0.001

Note: Asian, Asian or Pacific Islander; *AI/AN* American Indian/Alaska Native

Others^a^, Other specified parts of pancreas, Islets of Langerhans, Overlapping lesion of pancreas and Pancreas, NOS

Others^b^, Squamous cell carcinoma, Pancreatic endocrine tumor, Atypical carcinoid tumor, etc

### Construction and validation of the nomogram based on predictors of lymph nodes metastasis

**Fig. 2:**
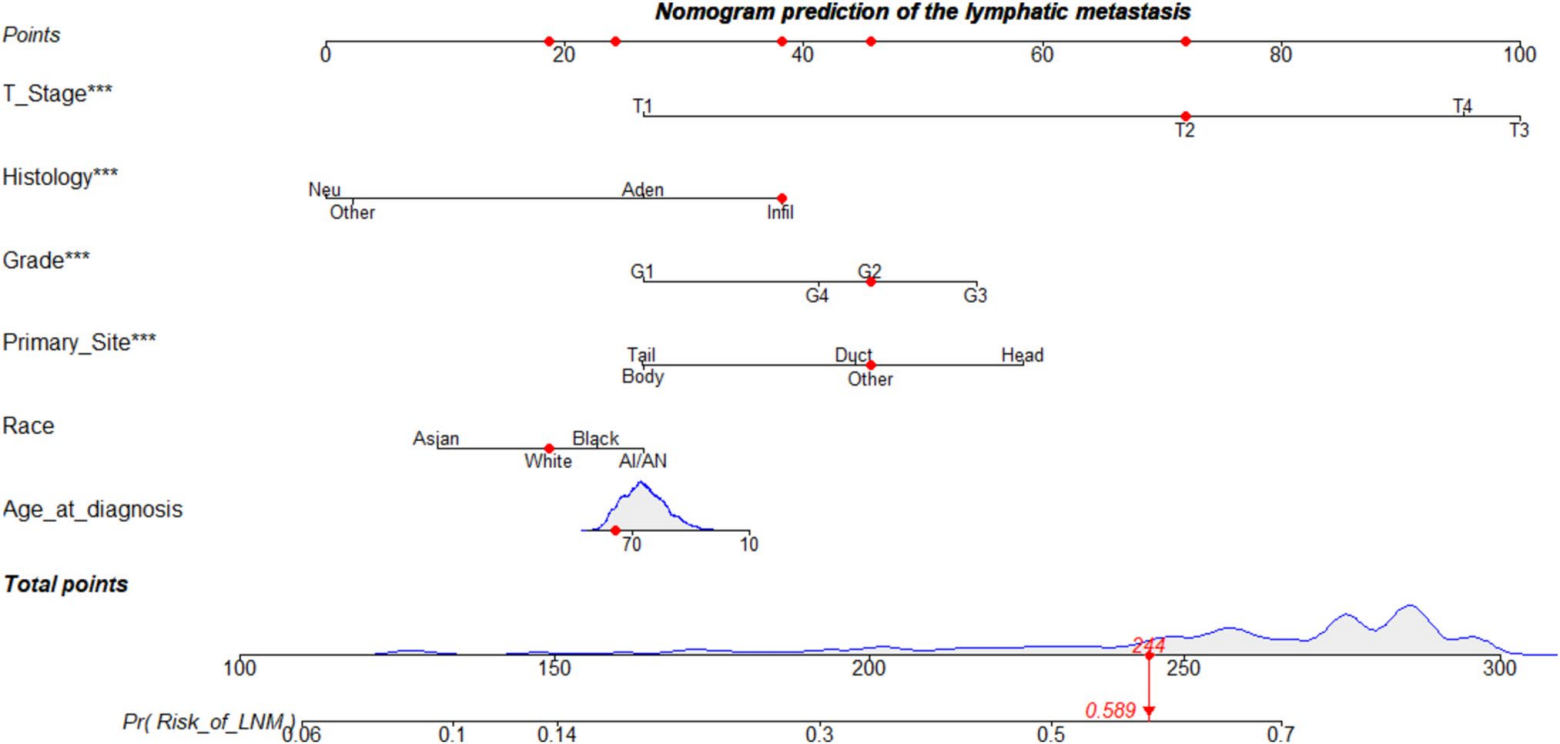
The nomogram for the risk of lymph node metastasis in resectable pancreatic cancer patients

**Fig. 3:**
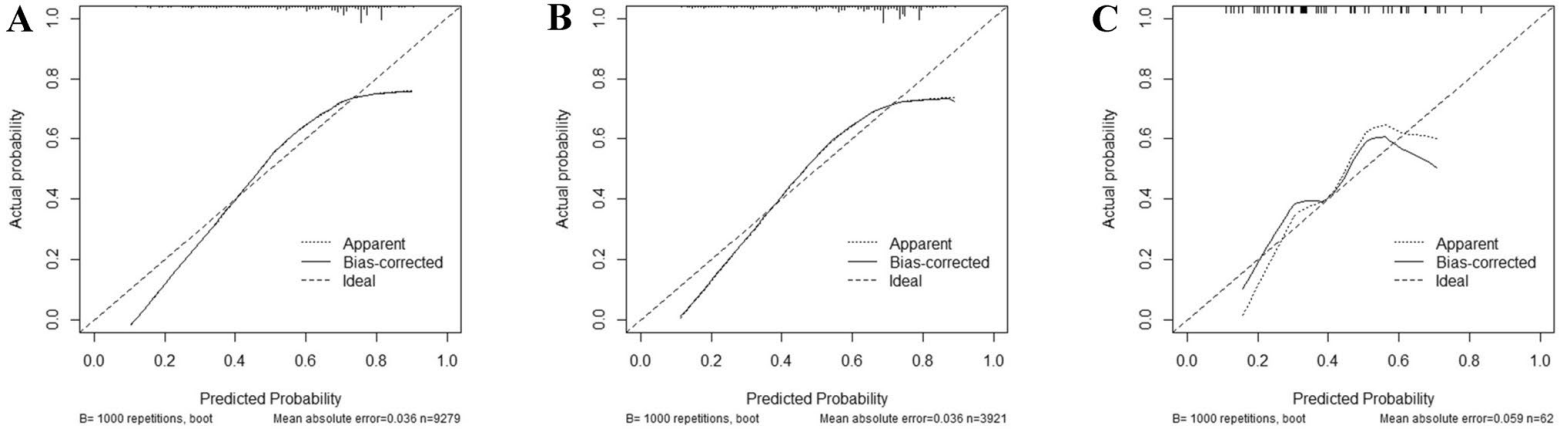
ROC of the nomogram for the training cohort (**A**), the internal validation cohort (**B**), and the external validation cohort (**C**)

**Fig.4:**
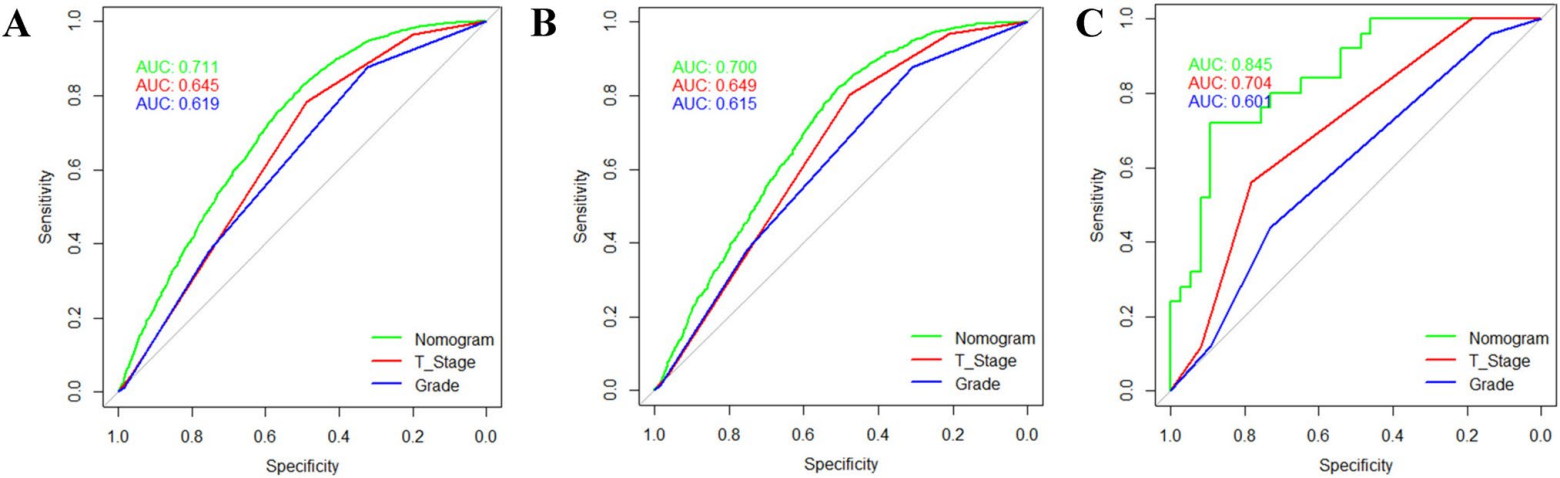
The calibration plots of the training cohort (**A**), the internal validation cohort (**B**), and the external validation cohort (**C**)

The minimum Akaike’s information criterion (AIC) was used to select the optimal model parameters and construct a nomogram for assessing the risk of LNM (Arunajadai 2009; Coles et al. 1980; Wang et al. 2004; Zhang 2016), and a total of six predictors including age at diagnosis, race, primary site, grade, histology, and T-stage were integrated to construct the nomogram (Fig. 2). The AUC was 0.711 (95%CI: 0.700–0.722) in the training, 0.700 (95%CI: 0.683–0.717) in the internal validation group, and 0.845 (95%CI: 0.749–0.942) in the external validation group, which proved a superior performance than the single factor (Fig. 3). The AUC of the T-stage and grade alone were lower than that of the nomogram. The AUC for T-stage was 0.645 (95%CI: 0.635–0.656), 0.649 (95%CI: 0.634–0.665), and 0.704 (95%CI: 0.587–0.821) in the training set, internal validation set and external validation set. Moreover, the AUC for the grade was 0.619 (95% CI: 0.608–0.630), 0.615 (95%CI: 0.598–0.632), and 0.601 (95%CI: 0.472–0.729) in the training, internal validation, and external validation groups, separately. Furthermore, the calibration plots show good consistency in the training set (C-index: 0.689), internal validation set (C-index: 0.686), and external validation set (C-index: 0.752) (Fig. 4). We also designed an online web calculator: https://xxlchxjh.shinyapps.io/DynNomappforLNMinpancreaticcancer/.

**Fig.5:**
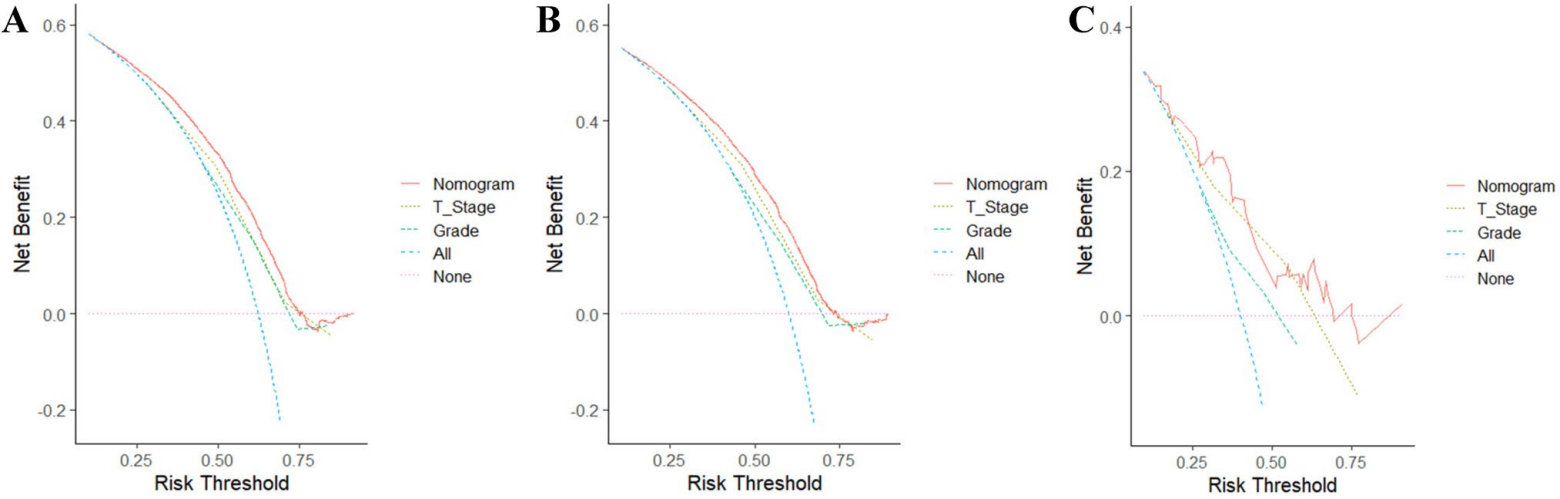
Nomogram decision curves (DCA) for the training cohort (**A**), the internal validation cohort (**B**), and the external validation cohort (**C**)

The clinical application value was determined by DCA which calculates the net benefits at different risk threshold probabilities. The net benefit of the nomogram was the largest in comparison to the grade and T-stage, which indicated the nomogram was a reliable clinical tool for predicting LNM in PC patients who underwent surgical resection (Fig. 5).

Additionally, the accuracy of the nomogram compared with the T-stage was demonstrated by the NRI and IDI. The NRI was 0.370 (95%CI: 0.329–0.411) and the IDI was 0.044 (95%CI: 0.039–0.048, *P* < 0.001) in the training group. The NRI and IDI in the internal validation group were 0.274 (95%CI: 0.211–0.337) was 0.035 (95%CI: 0.029–0.041, *P* < 0.001). In the external group, the NRI and IDI were 0.577 (95%CI: 0.091–1.063) was 0.062 (95%CI: 0.004–0.120, *P* = 0.037). The accuracy for predicting LNM by the nomogram was greater than the T-stage.

**Fig. 6:**
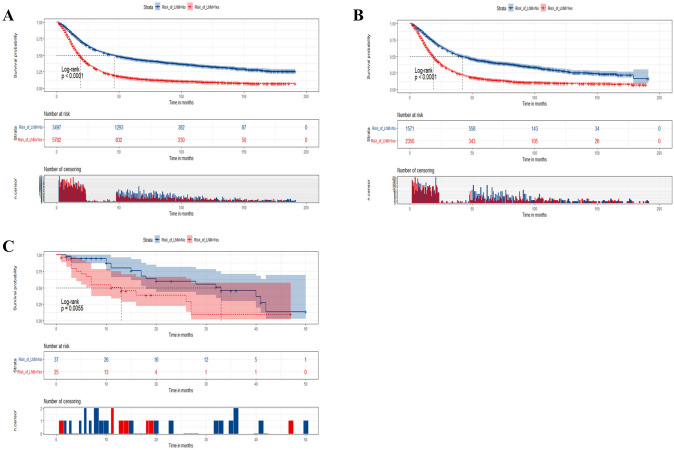
The Kaplan–Meier overall survival (OS) analysis of lymph node metastasis in the training set (**A**), the internal validation set (**B**), and the external validation set (**C**)

The Kaplan–Meier overall survival curves of training and internal/external validation groups are plotted in Fig. 6. The prognosis of PC patients with positive LNM was significantly lower in both training and internal/external validation groups. (*P* < 0.01).

## Discussion

PC is one of the most lethal of all cancers with high mortality, which is the seventh leading cause of cancer death worldwide (Sung et al. 2021). Even after surgical resection, early recurrence rates were reported to be 50% to 60%, with 5-year survival rates of only 20% to 30% (Gupta et al. 2017; Shin et al. 2018). PC patients with positive LNM have a worse prognosis with or without surgical resection. The status of LNM is a significant prognostic factor in PC patients, which is also important for the choice of treatment decisions. PC patients with positive lymph node metastasis should accept neoadjuvant chemotherapy or immunotherapy before surgical resection (Barrak et al. 2022; Kanda et al. 2011; Roland et al. 2015). Therefore, it is important to distinguish the status of lymph nodes before surgical resection in the clinic. At present, there are low sensitivities and specificities in evaluating lymph node metastasis by imageological examinations, and it is difficult to identify the LNM before surgical resection. Therefore, it is important to construct a sensitive and efficient prediction model for assessing the status of LNM preoperatively in PC patients.

In our study, a total of six clinicopathological factors were considered as risk factors associated with LNM in PC patients, including age at diagnosis, grade, histology, T-stage, primary site, and race, which was largely consistent with previous analyses (Huang et al. 2023; Song et al. 2018). The convenient preoperative nomogram prediction model was constructed by those independent predictors. This is the first research to construct and validate a nomogram for predicting LNM in resectable PC patients based on large populations. Previously, researchers pay more attention to the status of lymph node metastasis in pancreatic head cancer. Xingren Guo et al. developed a nomogram for predicting the lymphatic metastasis in pancreatic head cancer based on 191 pancreatic head cancer patients who received laparoscopic pancreaticoduodenectomy (Guo et al. 2023). Yi-Nan Shen et al. constructs a nomogram for predicting the peripancreatic vein invasion in pancreatic head cancer patients. Additionally, the other tumor sites of PC such as the body and tail of the pancreas also occur lymphatic metastasis (Shi et al. 2022; Tanaka et al. 2022, 2020), and a model for predicting the status of LNM in those tumor sites of PC is in need. The nomogram model constructed in our study could satisfy this requirement. In our study, it is obvious that PC patients with the tumor site in the head have more potential LNM compared with the tail and body of the pancreas, which was consistent with previous studies and clinical practice (Guo et al. 2023; Kobayashi et al. 2022).

Various studies demonstrated that race was related to lymph node metastasis and prognosis (Oweira et al. 2017; Zheng-Pywell et al. 2022). Rui Zheng-Pywell et al. reveals that black patients had a higher risk of LNM in tumors less than 2 cm in size compared with white patients (Zheng-Pywell et al. 2022). In our study, Asian PC patients such as Chinese, Japanese, and Korean were less likely to undergo LNM. Moreover, a higher positive rate of LNM was observed in black PC patients, which is consistent with the previous conclusion.

The correlation between grade and LNM in PC patients has been revealed in previous studies widely. Harimoto Norifumi et al. shows that lymph node metastasis was significantly associated with higher tumor grade in pancreatic neuroendocrine neoplasm (Harimoto et al. 2019). Similarly, our study found that grade was an independent risk factor associated with LNM in PC patients. LNM is more likely to occur in poorly differentiated or undifferentiated PC patients.

The histological type is commonly considered an important predictor of the prognosis in PC patients. Bi-Yang Cao et al. found that adenocarcinoma was the independently associated risk factor for poor prognosis in patients with liver metastasis in PC patients (Cao et al. 2023). Until now, there were few studies focused on the association between histological type and risk of LNM. In this study, there is a higher risk of LNM in PC patients with infiltrating duct carcinoma, while, PC patients with the histological type of neuroendocrine carcinoma have less LNM. Furthermore, the T-stage was a significant prognostic factor in PC, including the tumor size and infiltrating scope. In 2022, Xi-Tai Huang et al. showed that the T-stage was significantly associated with LNM in pancreatic neuroendocrine tumors (Huang et al. 2022). In this study, PC patients with T4 indicate more potential risk of LNM in comparison with T1 or T2.

The nomogram for evaluating the risk of LNM in PC patients was developed by easily available clinicopathological factors, including age at diagnosis, race, grade, histology, T-stage, and tumor location. The AUC and the calibration curves demonstrated excellent discrimination and consistency of this nomogram model. The risk of LNM in PC patients could be conveniently and accurately calculated by those accessible variables. Furthermore, DCA curves were utilized to estimate the clinical utility, which shows good net benefit. In summary, the risk of LNM in preoperative PC patients can be easily and accurately predicted by the newly established nomogram model.

Although the nomogram model had good accuracy for predicting the risk of LNM in PC patients, there are several limitations to this study. First of all, the selection bias could not be avoided due to the nature of retrospective analyses. For example, patients with missing data were excluded from our study, which may cause selection bias. Secondly, variables such as age, tumor size, leucocyte, albumin, and lymphocytes/monocytes have been identified as independent predictors of LNM in pancreatic head cancer (Guo et al. 2023). The serum CA 19–9, PC.ae.C42_5, and PC.aa.C38_4 were considered the powerful preoperative clinical variables in predicting the early recurrence of pancreatic cancer (Rho et al. 2019). However, those variables were not supplied in the SEER database. Therefore, those important variables cannot be incorporated into the nomogram model. Finally, the external validation data from our hospital are very little, which may lead to underfitting the model and more external validations are needed.

## Conclusion

In summary, the nomogram for predicting the preoperative LNM in PC patients was developed based on the SEER database, which shows good performance and clinical application.

## Data Availability

The data of this study are available for all authors.

## Abbreviations

PC: Pancreatic cancer
LNM: Lymph node metastasis
SEER: Surveillance, epidemiology, and end results
ROC: Receiver operating characteristic
AUC: Area under the curve
DCA: Decision curve analysis
CI: Confidential interval
OR: Odds ratios
IQR: Inter-quartile range
NRI: The nutrition risk index
IDI: Integrated discrimination improvement
